# FAM134B-mediated endoplasmic reticulum autophagy protects against cisplatin-induced spiral ganglion neuron damage

**DOI:** 10.3389/fphar.2024.1462421

**Published:** 2025-01-30

**Authors:** Fan Wang, Yue Xu, Yajie Wang, Qian Liu, Yanan Li, Weiwei Zhang, Huiming Nong, Junhong Zhang, Hao Zhao, Huaqian Yang, Lingchuan Guo, Jianfeng Li, Hong Li, Qianqian Yang

**Affiliations:** ^1^ Department of Otolaryngology-Head and Neck Surgery, Shandong Provincial Hospital, Shandong University, Jinan, China; ^2^ Department of Otolaryngology-Head and Neck Surgery, Shandong Provincial Hospital Affiliated to Shandong First Medical University, Jinan, China; ^3^ Department of Otolaryngology, The First Affiliated Hospital of Soochow University, Suzhou, China; ^4^ Department of Otolaryngology, Head and Neck Surgery, People’s Hospital, Peking University, Beijing, China; ^5^ Cyrus Tang Medical Institute, Soochow University, Suzhou, China; ^6^ Department of Pathology, The First Affiliated Hospital of Soochow University, Suzhou, China; ^7^ Shandong Provincial Key Laboratory of Otology, Jinan, China

**Keywords:** FAM134B, SGNS, cisplatin, ER-phagy, ER stress

## Abstract

**Introduction:**

Cochlear spiral ganglion neurons (SGNs) could be damaged by ototoxic drug, cisplatin (Cis), during which process autophagy was involved. FAM134B, the first detected endoplasmic reticulum autophagy (ER-phagy) receptor, plays an important part in the dynamic remodelling of the ER, the mutation of which affects sensory and autonomic neurons. However whether FAM134B-mediated ER-phagy involved in Cis-induced SGN damage or not was unknown. The present study was designed to determine whether FAM134B is expressed in SGNs of C57BL/6 mice and, if so, to explore the potential function of FAM134B in Cis-induced SGN damage *in vitro*.

**Methods:**

Middle turns of neonatal murine cochleae were cultured and treated with 30 μM Cis *in vitro*. The distribution of FAM134B, morphological changes of SGNs, and the colocalization of ER segments with lysosomes were measured by immunofluorescence (IF). Apoptosis was measured by TUNEL staining. The expression of FAM134B, proteins associated with ER stress, autophagy and apoptosis was measured by western blot. The reactive oxygen specie (ROS) levels were evaluated by MitoSOX Red and 2′,7′-Dchlorodihydrofluorescein diacetate (DCFH-DA) probe. Anc80-*Fam134b* shRNA was used to knockdown the expression of FAM134B in SGNs.

**Results:**

We first found the expression of FAM134B in the cytoplasm of SGNs, especially in the fourth postnatal day mice. Results showed decreases in the number of SGNs and FAM134B expression, as well as increases of ROS level, ER stress, ER-phagy, and apoptosis after Cis stimulus. Inhibiting autophagy increased the expression of FAM134B, and aggravated Cis-induced SGN damage, while the opposite changes were observed when autophagy was activated. Additionally, co-treatment with the N-Acetyl-L-Cysteine (NAC), ROS scavenger, alleviated Cis-induced ER stress, ER-phagy, and apoptosis. What’s more, knockdown the expression of FAM134B in SGNs made SGNs more vulnerable to cisplatin-induced injury.

**Discussion:**

The present study revealed the expression pattern of FAM134B in C57BL/6 murine SGNs for the first time. Moreover, our work further verified the protective function of FAM134B mediated by ER-phagy in Cis-induced SGN apoptosis, at least partially, correlated with the accumulation of ROS and induction of ER stress, though the detailed regulatory mechanism through which needs much more work to reveal.

## Introduction

Hearing loss is the most common sensory disorder, which not only seriously affects people’s health, causes depression, but also aggravates the global public health burden (Zhao et al., 2022). Cochlear spiral ganglion neurons (SGNs), responsible for converting mechanical signals from inner-ear hair cells (HCs) into electrical signals and transmitting to the brainstem, are essential for the formation of hearing (Zhao et al., 2022). Structural cellular loss or dysfunction of SGNs leads to sensorineural hearing loss (SNHL), the primary type of deafness (Ren et al., 2022). Given its non-renewable nature, deafness caused by the injury of SGNs is progressive and irreversible (Paken et al., 2019; Kurihara et al., 2022). It has been documented that multiple factors, including noise over-exposure, genetic disorders, aging, and ototoxic medications, target and cause impairments of SGNs. Among these factors, cisplatin (Cis) is a chemotherapy drug widely used in treatment of numerous solid malignant tumors in clinic with serious side effects (Laurell, 2019). Among its side effects, ototoxicity presents a significant challenge (Meijer et al., 2022) due to being short of effective treatment options for that the precise mechanisms has not been fully understood (Chen et al., 2022). It is exciting the advent of sodium thiosulfate (STS) as a preventative agent to reduce Cis-induced hearing loss (CIHL) by inhibiting intracellular reactive oxygen specie (ROS) formation and inactivation of cisplatin, however, not to be overlooked, it also has some limitations: 1) adverse events, such as nausea and vomiting, nephrotoxicity, neutropenia, and so forth; and 2) not suitable for patients with metastatic tumours; and 3) difficulty in the determination of the appropriate STS administration-time window for the variation of Cis treatment around the world (Meijer et al., 2024). Besides, the restoration of hearing loss caused by Cis has proven to be ineffective, highlighting the importance of further research into the complex mechanisms under CIHL. While the exact mechanisms remain incompletely elucidated, evidence suggests that the accumulation of ROS (Yu et al., 2020), autophagy, and apoptosis triggered by endoplasmic reticulum (ER) damage (Xu et al., 2014) are confirmed to be the plausible causes. ER is the largest membrane-bound organelle in mammalian cell, which plays an important role in the quality control of newly synthesized proteins by unfolded protein response (UPR) and the ER-associated protein degradation pathway (ERAD) (Mo et al., 2020). Endoplasmic reticulum autophagy (ER-phagy) is an other important and highly selective degradation form mediated by special autophagy receptors, timely clearing damaged ER fragments and ER-resident proteins via lysosomes by interacting with autophagosome-associated LC3, both under basal conditions and ER stresses (Cai et al., 2016; Grumati et al., 2018), thereby maintaining ER homeostasis (Bhaskara et al., 2019). Numerous experiments have shown the dual effects of ER stress on autophagy, that is, ER stress could induce autophagy to rescue slightly damaged cell components (Wang and Kaufman, 2012), whereas, excessive and prolonged ER stress will also activate apoptosis to clear badly damaged cells (Liu and Kaufman, 2003), revealing the central regulatory role of ER-phagy between initial stresses and the final cell fate.

Of the several known ER-phagy receptors, FAM134B (Alias: JK-1, RETREG1) is the first one discovered and belongs to protein family with sequence similarity 134 (FAM134), containing 497 amino acids encoded by the *FAM134B* gene located in the chromosomal region 5p (Mo et al., 2020). Importantly, the protein contains two hydrophobic structural domains, the LC3-interacting domain (LIR) and the reticulon-homology domain (RHD). The LIR domain is primarily responsible for binding to the autophagy protein LC3/GABARAP, and RHD domain is responsible for inducing ER membrane remodeling through its hairpin structure formed by two transmembrane fragments. The special structures focus FAM134B on its most powerful and famous ER-phagy regulatory function, and ER-phagy is implicated in a lot of pathophysiological processes. Deficiency of FAM134B can lead to ER expansion and activate ER stress, taking part in the formation and progression of numerous diseases. On the contrary, overexpression of FAM134B can promote the formation of autophagosomes, including reshuffling and inducing fragmentation, of ER membranes, which promote the turnover of damaged and dysfunctional ER (Khaminets et al., 2015). Not to be overlooked, a study has demonstrated that excessive ER-phagy mediated by FAM134B impaired the ER homeostasis, caused ER stress, and ultimately led to cell death in Hela cells (Liao et al., 2019), indicating the complicated regulation of FAM134B. Moreover, recent paper revealed its special function in sensory and autonomic neurons (Foronda et al., 2023). However, best to our knowledge, there was no researches about functions of FAM134B in auditory SGNs till now. Here we applied autophagy agonist, RAPA, and inhibitor, 3-MA (Zeng et al., 2023), to regulate the level of autophagy, during which process FAM134B-mediated ER-phagy has also been regulated at the same time, to research the possible function of FAM134B-mediated ER-phagy in Cis-related SGN damage. What’s more, we verified the protective function of FAM134B-mediated ER-phagy against cisplatin-induced SGN damage by knockdown the expression of FAM134B in SGNs using Anc80-*Fam134b* shRNA.

Of note, CIHL involves damages of both mitochondria and ER. Moreover, a tight connection among FAM134B-mediated ER-phagy, ER stress and cell apoptosis was verified in our previous paper in auditory HCs (Yang et al., 2023), revealing different cell organelles and signal pathways functioned as a whole exposing to stresses. However, whether a link exists between FAM134B and Cis-induced ototoxicity in SGNs or not has not been explored yet. Here, we aimed to explore the expression of FAM134B in SGNs and conduct preliminary study on Cis-induced SGN damage, mainly focused on its ER-phagy receptor function, trying to provide novel insights into the complex mechanism of CIHL.

## Materials and methods

### Experimental animals

Wild-type (WT) C57BL/6 mice at different ages of postnatal day 4, 1 month, 4 months and 12 months were purchased from Jinan Pengyue Experimental Co. (Jinan, China). All animal procedures were performed according to protocols approved by the Animal Care Committee of Shandong University and Animal Experimental Ethical Inspection Form of Shandong Provincial Hospital, Jinan, P.R. China (NO. 2022-141) and were consistent with the National Research Council’s Guide for the Care and Use of Laboratory Animals. All efforts were made to reduce their suffering.

### Culture of neonatal cochlear SGNs explants

**FIGURE 1:**
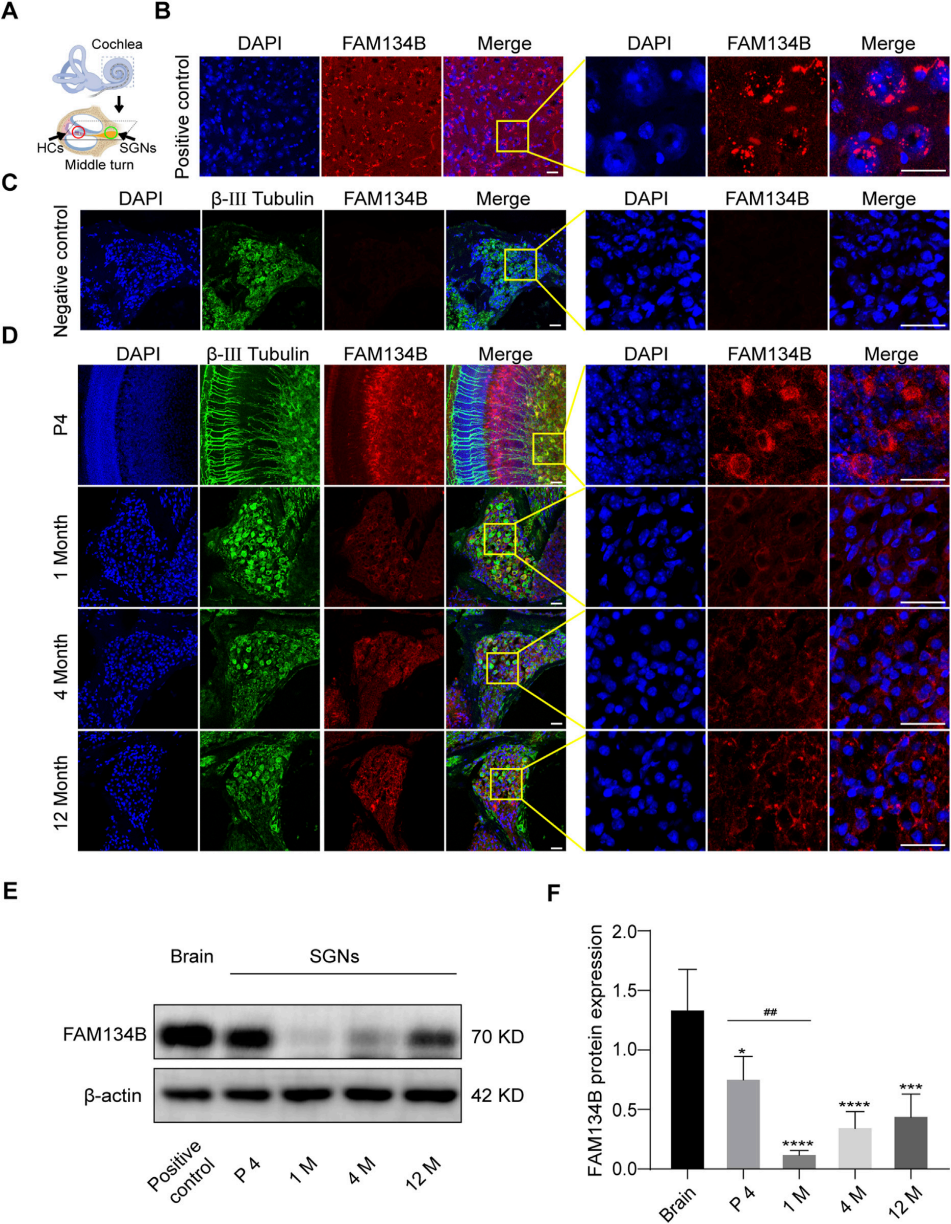
FAM134B is expressed in cochlear SGNs of C57BL/6 mice. **(A)** Pattern diagram of cochlea and the middle turn. Red circle showed organ of Corti, where HCs located. Green circle showed cochlear axis where SGNs located. **(B)** Positive control: FAM134B was widely expressed in cytoplasm of brain cells mainly around the nuclei corresponding to the same distribution of ER network. **(C)** Negative control: no FAM134B primary antibody was added in the immunostaining process. **(D)** The expression of FAM134B in cochlear SGNs of P4, 1-month old, 4-month old and 12-month old C57BL/6 mice. IF results showed that FAM134B was expressed in the cytoplasm of SGNs, which was similar to the expression pattern in the brain. FAM134B expression was more pronounced at the age of P4 in SGNs compared with other ages. Scale bars = 25 µm. **(E, F)** WB verified the relative expression of FAM134B in SGNs and brain, n = 3. ^##^p < 0.01 vs. SGNs of P4, *p < 0.05, ***p < 0.001, ****p < 0.0001 vs. brain tissue.

C57BL/6 mice at P4 were decapitated after anesthesia. The skull was divided into two-halves along the midline after removing the skin, and transferred into a Petri dish with ice-cold PBS. The cochlea was carefully dissected with fine anatomical forceps, and then the stria vascularis was gradually taken out, leaving the middle turn of SGNs with the basal membrane to keep the nerve fibers undamaged (Figure 1A). The middle turn of the SGNs then was pasted onto 10-mm coverslips precoated with Cell-TaK (354241, Corning) and incubated in Dulbecco’s Modified Eagle Medium/F12 (DMEM/F12; 11,330,032, Gibco) supplemented with EGF, FGF, etc. at 37°C in a 5% CO_2_ atmosphere.

### Drug treatments

After culturing the ganglion explants overnight, the medium was replaced. We separately placed the samples into well plates containing 0 μM, 10 μM, 30 μM, 50 μM cisplatin for 0 h, 6 h, 12 h, 24 h. The degree of damage to the SGNs was moderate when the SGNs were treated with 30 μM cisplatin for 24 h, which was the optimal treatment condition chosen for the following experiments. In addition, we pretreated SGNs with autophagy agonist (rapamycin, RAPA) (0.1 μM; V900930, Sigma-Aldrich) or inhibitor (3-methyladenine, 3-MA) (5 mM; 189490, Sigma-Aldrich) for 6 h, pretreated with ROS inhibitors (N-Acetyl-L-Cysteine, NAC) (2 mM; A7250, Sigma-Aldrich) for 2 h, and then co-administered with cisplatin for 24 h to determine the function of autophagy and ROS in cisplatin-induced ototoxicity, the concentrations of which were used as previously reported (Liu et al., 2021).

### Protein extraction and Western blot (WB)

The SGNs treated with drugs were placed in a small dish containing frozen PBS. Subsequently, the basement membranes were carefully removed with fine dissecting tweezers under the microscope, and only the SGNs were left. The tissues were broken by ultrasonic crusher and then extracted with RIPA lysate (R0020, Solarbio) containing protease inhibitors (p0100, Solarbio) and phosphatase inhibitors (HYK0022, MCE), centrifuged at 12,000 × g, 4°C for 30 min, and the supernatant was collected. The protein concentration of the samples was determined by BCA protein determination kit (pc0020, Solarbio), and the protein was denatured in equal amounts. Then, the protein was separated by 10% or 12% SDS-PAGE electrophoresis and transferred to polyvinylidene fluoride (PVDF) (ISEQ00010, Merck Millipore) membrane. The membrane was sealed with 5% BSA or 5% skimmed milk at room temperature for 1 h, and then incubated with the diluent of the relevant primary antibody at 4°C overnight. On the second day, the membrane was washed three times with TBST and then incubated with the relevant secondary antibody dilutions for 1 h at room temperature. Finally, ECL Kit (WBKLS0100, Millipore) was used to detect and ImageJ software was used to analyze the results. Primary antibodies were used as follows: anti-FAM134B antibody (1:1000, 83414S, CST), anti-LC3B antibody (1:1000, AB48394, Abcam), anti-cleaved caspase-3 antibody (1:1000, 9664S, CST), anti-caspase-12 antibody (1:1000, 35965S, CST), anti-Bcl-2 antibody (1:1000, A19693, Abclonal), anti-P-IRE1α antibody (1:1000, AB124945, Abcam), and anti-β-actin antibody (1:1000, GB11001, Servicbio).

### MitoSOX red staining

MitoSOX Red (M36008, Invitrogen) was used to assess mitochondrial ROS. The SGNs were washed with preheated PBS after treatment with drugs and incubated with 5 μM MitoSOX Red at 37°C for 10 min. The stained SGNs were observed and imaged with confocal microscope. The whole process needs to be protected from light.

### DCFH-DA staining

After drug treatment, the SGNs were washed with PBS. DMEM containing 10 μM DCFH-DA (without serum) (D6883, Sigma) was added to the tissues. They were placed in a light-proof box and subsequently incubated for 30 min in an incubator at 37°C. Then the tissues were washed with PBS for 3 times, each time for 5 min. The stained SGNs were placed and observed under the confocal laser microscope.

### Frozen section

1-month-old, 4-month-old and 12-month-old wild type C57BL/6 mice were decapitated after anesthesia. Their inner ears and brains were taken out under microscope to fix in 4% paraformaldehyde (PFA) overnight. The inner ears had to be decalcified in 0.5 M EDTA for at least 6 h on the shakers. Then, the decalcified inner ears and the fixed brain tissue were dehydrated with 15%, 20% and 30% sucrose in PBS, embedded in OCT gel and cut into slices at the thickness of 7–10 μm.

### TUNEL staining

Click-iT^®^ Plus TUNEL Assay (C10619, Life technologies) was used to examine the apoptotic levels of different groups of SGNs *in vitro* according to the manufacturer’s instructions. Which can be followed by other staining such as DAPI and other needed antibodies. The results were visualized by a Leica TCS SP8 confocal fluorescence microscope (Leica Microsystems, Biberach, Germany).

### Immunofluorescence staining

The SGNs were fixed with 4% PFA at room temperature for 30 min, then permeated with PBS containing 1% Triton X-100 for 30 min and immersed in blocking solution (0.1% Triton X-100, 5% inactivated donkey serum (D9663, Sigma-Aldrich), 1% bovine serum albumin (A1933, Sigma-Aldrich) in PBS at room temperature for 1 h. Next, the SGNs were incubated with the primary antibody at 4°C overnight. The main antibodies used in this study were anti-β-III tubulin antibody (1:400, 801201, Biolegend), anti-FAM134B antibody (1:200, 83414S, CST), anti-LC3B antibody (1:800, AB48394, Abcam), anti-cleaved caspase-3 antibody (1:1000, 9664S, CST), anti-caspase-12 antibody (1:200, 55238-1-AP, Proteintech), anti-Lamp1 antibody (1:100, MA1-164, Invitrogen), and anti-Calnexin antibody (1:800, AB22595, Abcam). Then, after washing them three times by PBS, they were incubated with secondary fluorescent antibody and DAPI (d9542, Sigma-Aldrich, United States) in the dark for 1 h. The slides were finally mounted and the specimens were observed and imaged using a Leica TCS SP8 confocal fluorescence microscope (Leica Microsystems, Biberach, Germany). C57BL/6 brain tissue was set as a positive control (Figure 1B), and SGNs without FAM134B primary antibody was used as a negative control (Figure 1C).

### Co-localization analyses

The captured confocal images were processed by ImageJ. Iso-surfaces were generated to localize the region of analysis using the red (Calnexin) and green (Lamp1) channel. The color threshold was set to a uniform value to ensure experimental accuracy. The processed images were analyzed by co-localization finder plugin and co-localized scatter plots were generated. Pearson correlation coefficient was subsequently obtained. Next, ImageJ was used to measure the percentage of the overlap area of the red channel (Calnexin) and the green channel (Lamp1) in the total area for statistical analysis.

### Counts for SGNs

Images were processed and analyzed using ImageJ. We used DAPI for nuclear staining, and the intact SGN nuclei were large and round. The number of damaged neuronal nuclei and the number of total SGN nuclei were counted manually, and the proportion of damaged nuclei was finally obtained. We used β-III tubulin for specific labeling of neurons. The number of β-III tubulin-positive SGN cells and nerve fibers were manually counted. The area of Rosenthal’s canal was also measured. Finally, the number of SGNs was divided by the area of Rosenthal’s canal using software to obtain the SGN cell and nerve fiber density per unit area.

### FAM134B knockdown by use of Anc80-*Fam134b* shRNA

To verified the specific role of FAM134B during cisplatin-induced SGN damage, we conducted *Fam134b* specific interfering adeno-associated virus (AAV) vectors purchased from OBiO Technology (Shanghai, China). AAV vector Anc80L65 (Anc80) carried shRNA sequences targeting mouse *Fam134b*. We obtained three different shRNA sequences:Anc80-*Fam134b*-shRNA-GFP-1, Anc80-*Fam134b*-shRNA-GFP-2, Anc80-*Fam134b*-shRNA-GFP-3. Cultured cochlear SGNs were separately treated with 4 × 10^11^ GC mL^−1^ AAV vectors described above for 24 h. The culture was continued for 24 h after replacement with new medium. Cisplatin was added to continue treatment for another 24 h after AAV-mediated transfection into the SGN with 48 h. Subsequently, Western blot analysis and immunofluorescence staining were performed. The specific sequences were listed in Table 1.

**TABLE 1:** ShRNA sequence.

Marker	Gene	Gene ID	TargetSeq
ShRNA-control	NC	NA	TTC​TCC​GAA​CGT​GTC​ACG​T
ShRNA-1	Retreg-1	NM_001034851.2	CAG​CTA​CCT​TCT​GTT​ACT​GTT
ShRNA-2	Retreg-1	NM_001034851.2	CAC​AAG​GAT​GAC​AGT​GAA​TTA
ShRNA-3	Retreg-1	NM_001034851.2	AGT​CAA​GTC​CAT​TCT​ATT​AAA

### Statistical analysis

Statistical analysis was performed using GraphPad Prism 8 software. Data were presented as mean ± SD of at least three separate experiments. Different analysis methods were selected according to the experimental data. Two-sided unpaired *t*-test was used to analyze two groups of data, and one-way ANOVA and Dunnett’s multiple comparison tests were employed to analyze two or more data groups. P < 0.05 was considered statistically significant.

## Results

### FAM134B is expressed in cochlear SGNs of C57BL/6 mice

We dissected the middle turn from the cochlea of the P4 C57BL/6 mice (Figure 1A) and first examined the expression pattern of FAM134B in the cochlear ganglion of C57BL/6 mice at different ages by use of IF and WB. IF showed that FAM134B was widely expressed in the cytoplasm of SGNs (Figure 1D). In addition, WB verified the highest expression of FAM134B in murine SGNs at the age of P4, and the significant decrease at 1 month. Compared with the age of P4, there was no significant difference in FAM134B expression at the age of 4 months and 12 months (Figures 1E, F).

### Cis induced SGN damage *in vitro*

**FIGURE 2:**
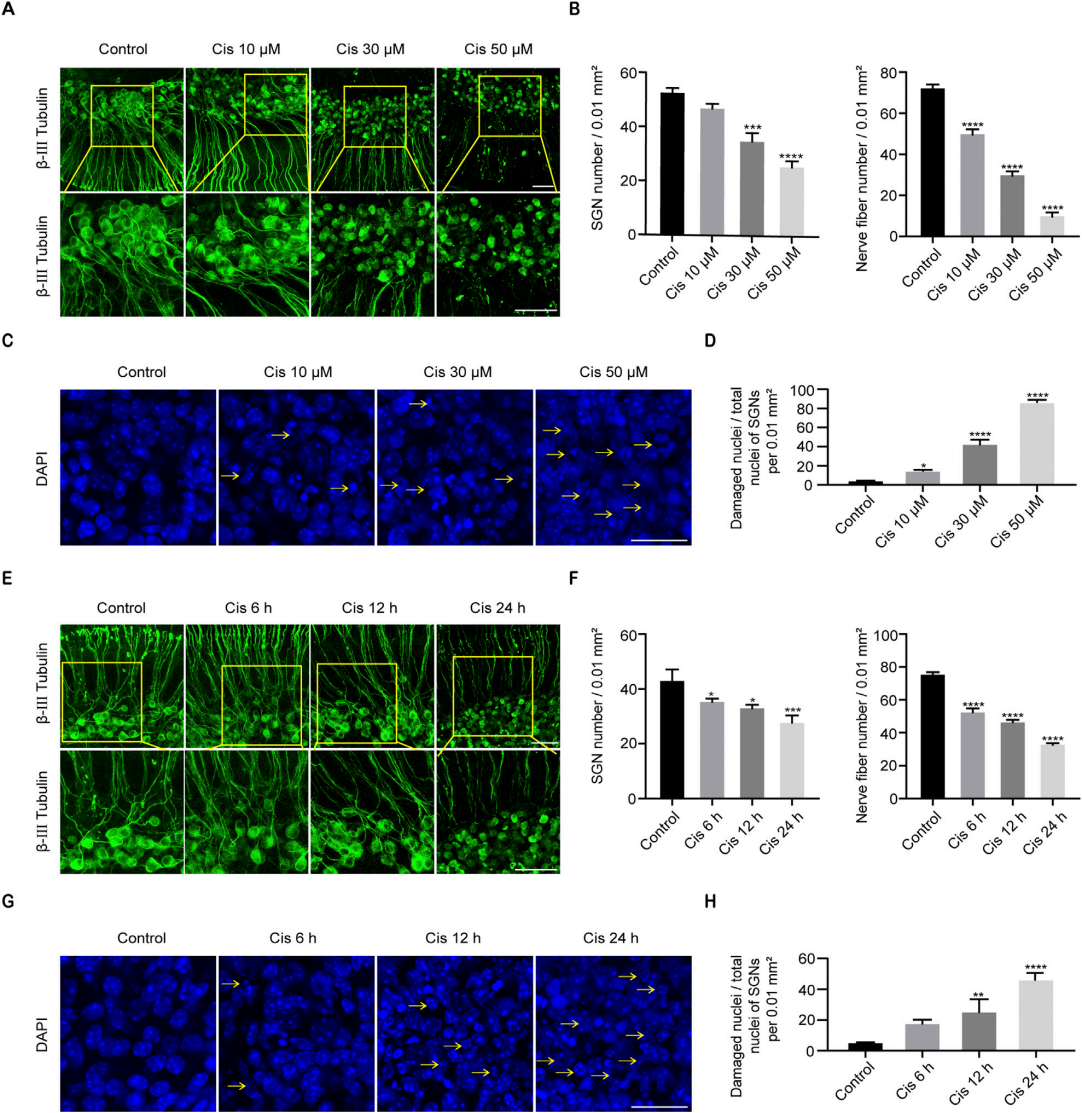
Cisplatin induced damage in SGNs. **(A, B)** Morphological changes, damage degree reflected by numbers of SGNs per 0.01 mm^2^ and nerve fibers per 0.02 mm^2^ after stimulus of Cis at different concentrations. β-III tubulin (green fluorescence) was served as a marker for cochlear spiral ganglion neurons. Scale bars = 50 μm, n = 3. **(C, D)** Morphological changes and ratio of damaged nuclei in SGNs under treatment of different concentrations of Cis were presented (arrows: damaged nuclei). Scale bars = 25 μm, n = 3. **(E, F)** Morphology and number of remaining SGN and nerve fibers after stimulus of 30 μM Cis for different times (6 h, 12 h and 24 h). Scale bars = 50 μm, n = 3. **(G, H)** Morphology and ratio of damaged nuclei of SGNs treated with 30 μM Cis for different times. Scale bars = 25 μm, n = 3. *p < 0.05, **p < 0.01, ***p < 0.001, ****p < 0.0001 vs. control group.

The middle turns of P4 C57BL/6 mice were taken out for culture, and then Cis was added on the second day. Culture media containing 10 μM, 30 μM or 50 μM Cis were applied to treat the samples for 24 h. And 30 μM Cis was used for 6 h, 12 h and 24 h, and the changes were compared with the control group. IF showed that the cellular morphology of SGNs in the control group was oval and large in size, the nuclei were intact, and the nerve fibers were arranged in a regular pattern. After treatment with Cis, results exhibited morphological characteristics of damage, such as the contraction of cytosol, the fragmentation and shrinkage of nuclei, and the destruction and depletion of nerve fibers (Figures 2A, C, E, G), and the damage degree was elevated as the Cis concentration increased and the action time was prolonged. The number of SGNs per 0.1 mm^2^ and nerve fibers per 0.2 mm^2^, and the proportion of damaged SGN nuclei compared to total SGN nuclei after treatment of Cis were quantified (Figures 2B, D, F, H). The damage degree of SGNs when treated with 30 μM cisplatin for 24 h was moderate, which was the optimal treatment condition chosen for the following experiments.

### Cis *elicited* apoptosis in C57BL/6 murine cochlear SGNs *in vitro*

**FIGURE 3:**
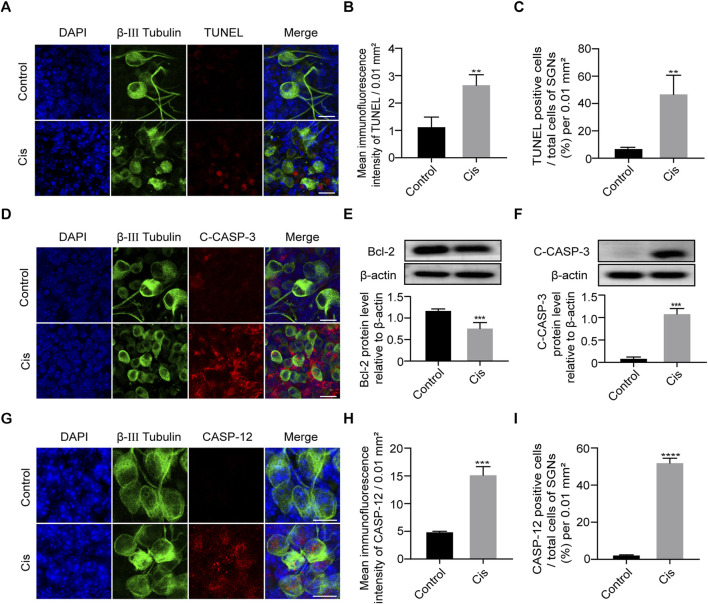
Cisplatin induced apoptosis in SGNs. **(A–C)** Representative images and analyses of TUNEL staining (red fluorescence) of SGNs after 24 h of cisplatin exposure with 30 µM. TUNEL immunofluorescence intensity and the number of TUNEL positive cells increased after Cis exposure for 24 h. Scale bars = 15 μm, n = 3. **(D)** Co-staining immunofluorescence of cleaved caspase-3 (red) and β-III tubulin (green fluorescence). Immunofluorescence showed that caspase-3 was activated to form cleaved caspase-3 (red fluorescence) after 24 h treatment with Cis. Scale bars = 15 µm. **(E, F)** Western blot showed that the expression level of cleaved caspase-3 was higher, but bcl-2 was lower in Cis-treated group, n = 3. **(G–I)** Co-staining immunofluorescence of caspase-12 (red fluorescence) and β-III tubulin (green fluorescence). Immunofluorescence intensity of the caspase-12 and the number of caspase-12 positive SGN cells increased after Cis exposure. Scale bars = 15 μm, n = 3. **p < 0.01, ***p < 0.001, ****p < 0.0001 vs. control group.

As apoptosis is the primary cell-death mode of cisplatin-induced ototoxicity, we next verified the SGN cell-death type focused mainly on apoptosis (Qiao et al., 2024). Results showed that cell apoptotic level significantly increased after Cis treatment for 24 h compared with control group (Figures 3A–C). Meanwhile, IF and WB showed that caspase 3 was significantly activated (C-CASP-3) and anti-apoptotic protein Bcl-2 was decreased after Cis exposure (Figures 3D–F). The results described above verified that Cis induced mitochondrial apoptotic pathway in SGNs. In addition, IF intensity analysis revealed the increased expression of caspase 12, a key ER stress related apoptotic mediator, and the proportion of caspase-12 positive cells significantly increased after Cis treatment (Figures 3G–I), revealing the activation of ER-stress related apoptosis.

### Cis down-regulated the expression levels of FAM134B, activated ER tress and ER-phagy in SGNs

**FIGURE 4:**
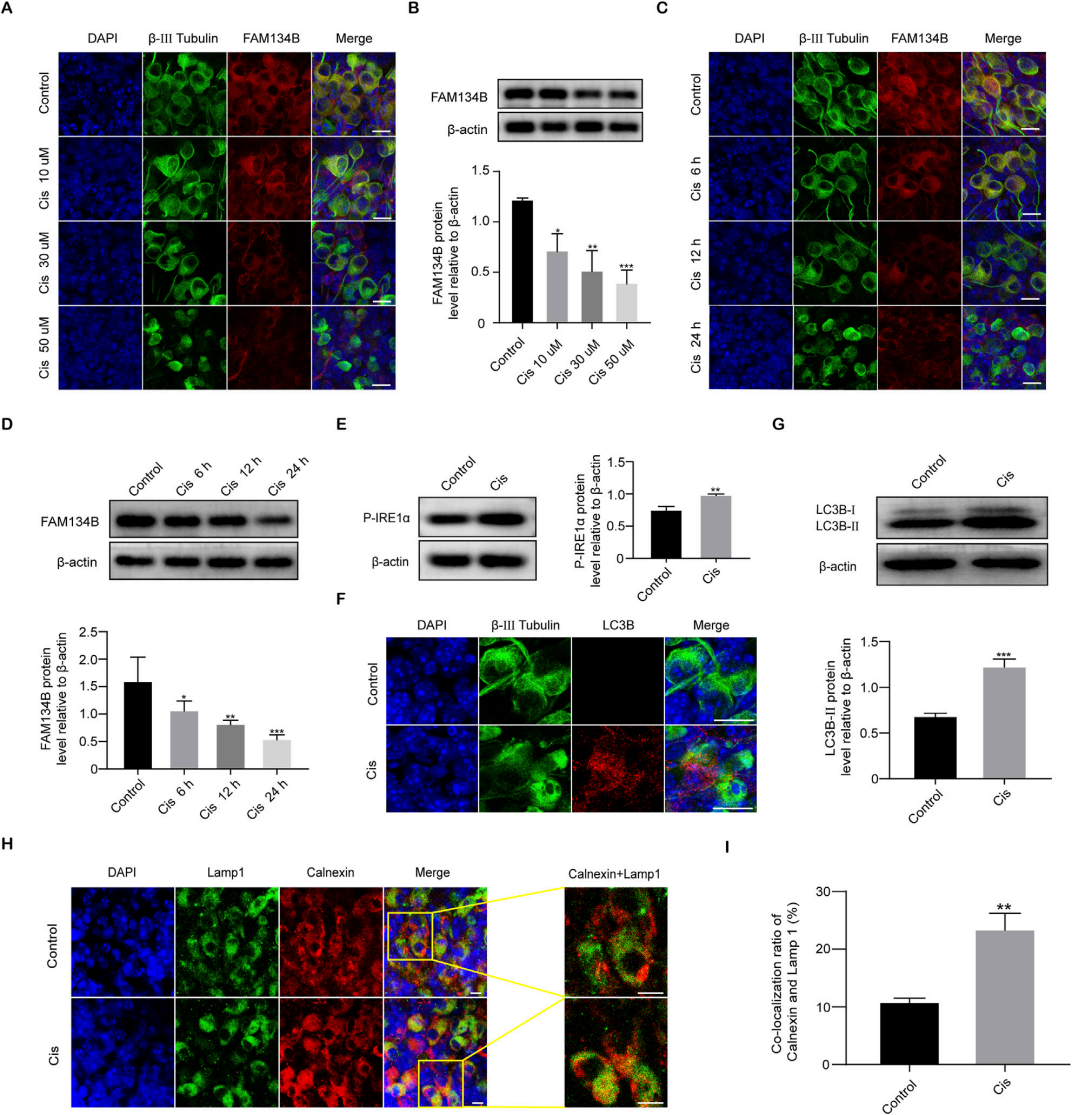
Cis induced a decrease in FAM134B expression and activated ER-phagy. **(A, B)** Staining of FAM134B (red fluorescence) and β-III tubulin (green fluorescence), a marker for SGNs. FAM134B expressed in SGNs and the expression level reduced progressively as Cis concentration increased and functional time prolonged. Scale bars = 15 μm, n = 3. **(C, D)** Western blot confirmed the decreased expression of FAM134B as the Cis concentration increased and functional time prolonged, n = 3. **(E)** Western blot results displayed the significant increase of P-IRE1α after 24 h stimulus with Cis, n = 3. **(F)** Immunofluorescence showed a significant growth in puncta of autophagy marker protein LC3B (red fluorescence) in response to cisplatin injury for 24 h. Scale bars = 15 µm. **(G)** Western blot showed that the expression level of LC3B-II in the Cis group was enhanced than that in the control group, n = 3. **(H, I)** Fluorescence colocalization of LAMP1 (green) with Calnexin (red). Immunofluorescence revealed that the ratio of colocalization grew up after Cis exposure for 24 h. Scale bars = 5 μm, n = 3. *p < 0.05, **p < 0.01, ***p < 0.001 vs. control group.

After the application of Cis, the IF intensity of FAM134B gradually decreased as the Cis concentration increased and time of stimulus prolonged (Figures 4A, B). Quantitative analysis by use of WB verified the decrease of FAM134B in a concentration- and time-dependent manner in response to Cis observed previously in IF measurement (Figures 4C, D). Under Cis-induced ER stress, eukaryotic cells respond through UPR to facilitate proper folding of the miss folded proteins mediated by functional proteins, such as Protein kinase RNA (PKR)-like ER kinase (PERK), activating transcription factor 6 (ATF6), and inositol-requiring kinase 1α (IRE1α) (Yang et al., 2023), so the increased level of which could serve as a reliable marker of ER stress. In our present work, Western blot showed increased expression of phospho-IRE1α (P-IRE1α), activated form of IRE1α, indicating the ER stress induced by Cis (Figure 4E). At the same time, the punctate expression of LC3B, an autophagy marker, was significantly increased in SGNs after treatment with Cis for 24 h compared with that in the control group (Figure 4F), which was verified by the following western blot (Figure 4G). Moreover, to verify specific ER-phagy, we further examined the colocalization of LAMP1, a marker of lysosomes, and Calnexin, a marker of ER, by IF. Results showed increased co-localization ratio of lysosomes and ER (Figures 4H, I), indicating the elevated ER-phagy in Cis-induced SGN damage.

### The expression levels of FAM134B changed oppositely with degrees of ER-phagy in SGNs after co-stimulation of autophagy regulators and Cis

**FIGURE 5:**
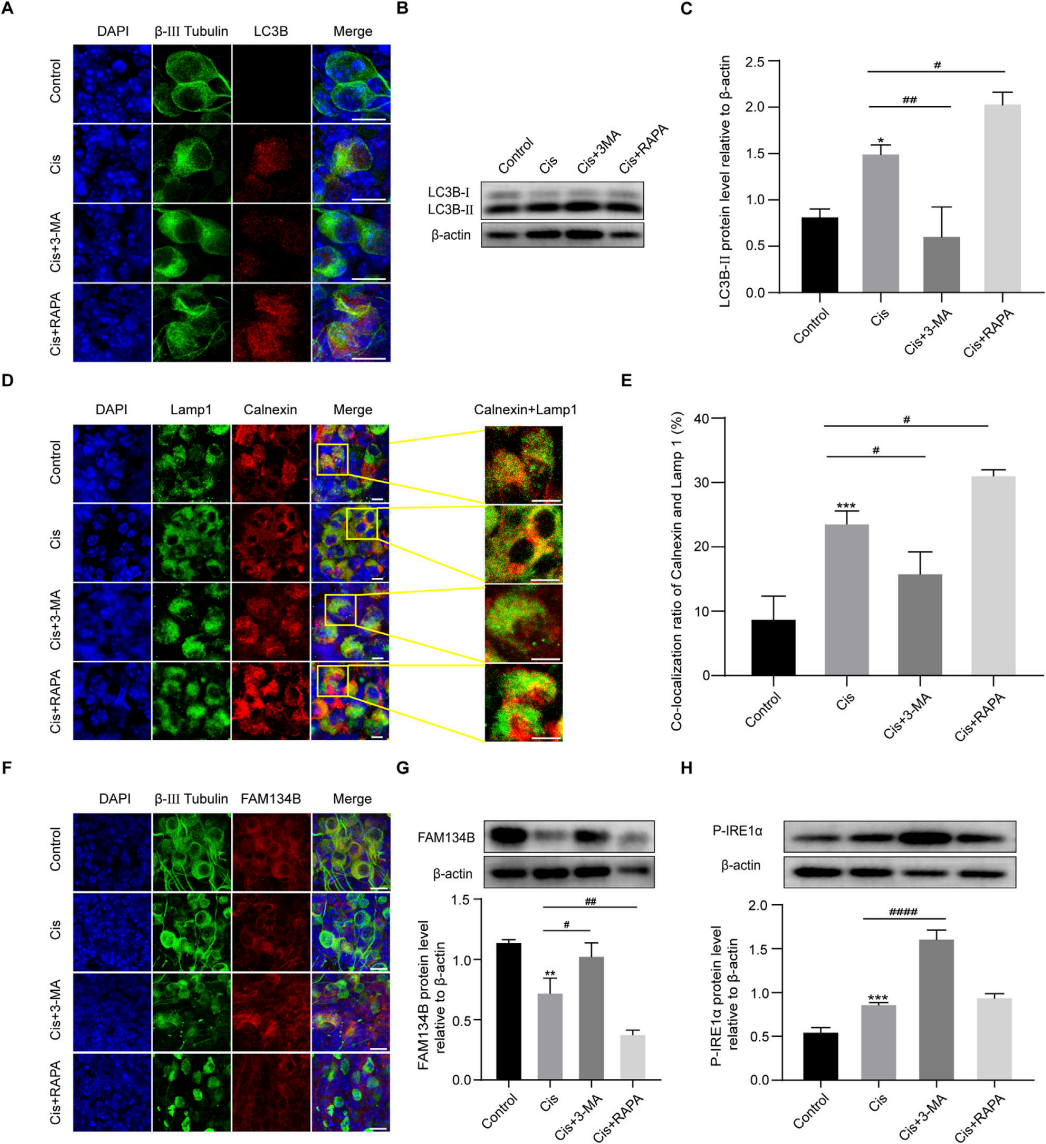
The expression levels of FAM134B changed oppositely with degrees of ER-phagy in SGNs after co-stimulation of autophagy regulators and Cis. **(A)** Immunofluorescence of LC3B (red) and β-III tubulin (green) showed an increased LC3B puncta in SGNs after cisplatin treatment for 24 h compared to control group. LC3B puncta were increased in Cis + RAPA group and decreased in Cis + 3 MA group compared to that in Cis group. Scale bars = 15 µm. **(B, C)** Western blot showed the quantification of LC3B-II expression after application of Cis and autophagic regulators, n = 3. **(D)** Fluorescence colocalization of LAMP1 (green) with Calnexin (red). Scale bars = 5 µm. **(E)** Proportion analysis of fluorescence co-localization, n = 3. **(F)** Co-staining of FAM134B (red) and β-III tubulin (green) revealed variation of FAM134B expression in SGNs collected from different groups. Scale bars = 15 µm. **(G)** Dynamic expression of FAM134B after the application of Cis and Cis + autophagic regulators were detected by Western blot, n = 3. **(H)** Dynamic changes in ER stress-related protein P-IRE1α expression in SGNs after 24 h stimulation with Cis or Cis + autophagic regulators. β-actin was used as a loading control, n = 3. The differences were statistically significant. ^#^p < 0.05, ^##^p < 0.01, ^####^p < 0.0001 vs. cis group, *p < 0.05, **p < 0.01, ***p < 0.001 vs. control group.

Given the above results that Cis injury increases ER-phagy in the cultured cochlear SGNs, and as the primary mediator of ER-phagy, the expression of FAM134B changed during the damage process, providing direct evidence of the involvement of FAM134B-mediated ER-phagy in Cis-induced SGN injury. To verify the functional correlation of FAM134B and autophagy during this process, we then regulated autophagy levels in SGNs, and observed possible changes of FAM134B expression by treating with autophagy activator, RAPA (100 nM), and autophagy inhibitor, 3-MA (5 mM), for 6 h before cotreatment with Cis for 24 h. The does of the regulators used here were selected according to the previous literature about SGNs *in vitro* (Liu et al., 2021). Immunofluorescence and Western blot showed increased LC3B punctate and LC3B-II expression in SGNs collected from autophagy-activated (Cis + RAPA) group compared with Cis group. We obtained the contrary changes in autophagy inhibited (Cis + 3-MA) group (Figures 5A–C), revealing that RAPA and 3-MA could effectively activate and inhibit autophagy in SGNs under cisplatin treatment respectively. Immunofluorescence also showed promoted ER-phagy, labeled by elevated ratio of co-localization of lysosomes (marked by LAMP1) and ER (marked by Calnexin) in autophagy-activated (Cis + RAPA) group, and the opposite results in Cis + 3-MA group compared with Cis group (Figures 5D, E). At this basis, we next examined the expression of FAM134B, which decreased in autophagy activated (Cis + RAPA) group, and increased in autophagy inhibited (Cis + 3-MA) group (Figures 5F, G). Moreover, ER stress levels during the process were also measured. The expression of P-IRE1α increased in autophagy-inhibited (Cis + 3-MA) group, but not changed obviously in autophagy-activated (Cis + RAPA) group (Figure 5H).

### Autophagy protect against Cis-induced SGN damage by relieving apoptosis

**FIGURE 6:**
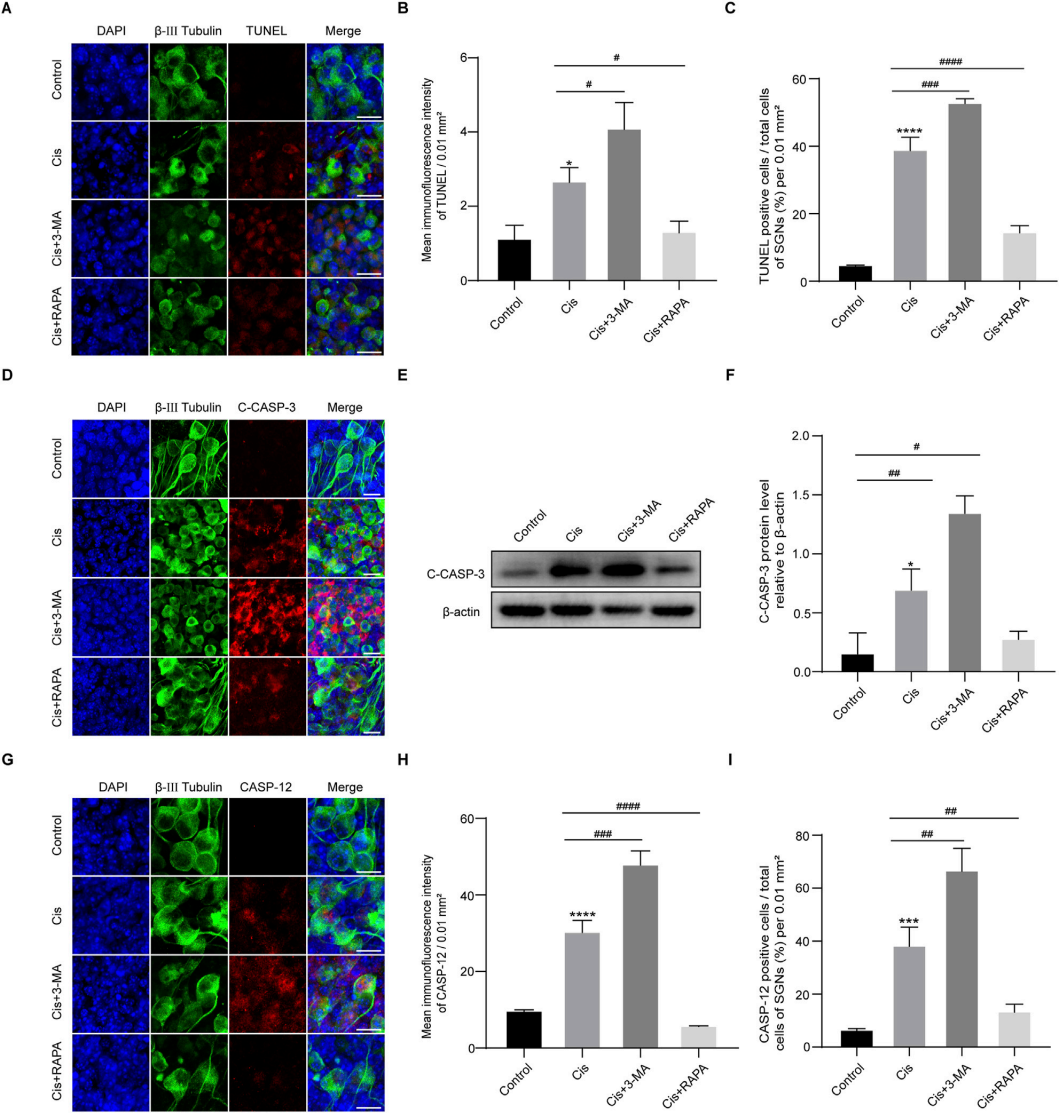
Induction of autophagy relieved Cis-induced SGN apoptosis. **(A–C)** Representative images and analyses of TUNEL staining (red fluorescence) of SGNs. The ratio of TUNEL-positive cells raised in 3-MA + Cis group, and declined in Cis + RAPA group compared with that in Cis group. Scale bars = 15 μm, n = 3. **(D)** Co-staining of cleaved caspase-3 (red) and β-III tubulin (green). Cleaved caspase-3 was sharply cut down in Cis + RAPA group and obviously increased in Cis + 3-MA group compared to the Cis-only group. Scale bars = 15 µm. **(E, F)** Analysis of dynamic changes of cleaved caspase-3 by WB, n = 3. **(G–I)** Analyses of caspase-12 (red) in SGNs (β-III tubulin positive, green) after application of 3-MA and RAPA. Scale bars = 15 μm, n = 3. The differences were statistically significant. ^#^p < 0.05, ^##^p < 0.01, ^###^p < 0.001, ^####^p < 0.0001 vs. cis group, *p < 0.05, ***p < 0.001, ****p < 0.001 vs. control group.

To verify the function of autophagy in SGN damage caused by Cis, we further detected cell apoptosis by labeling SGNs with TUNEL and measuring the variation of the key apoptotic mediators after regulation of autophagy. As we can see in Figure 6, autophagy inhibition (3-MA cotreatment) aggravated Cis-induced SGN apoptosis, reflected by upregulated TUNEL positive puncta, cleaved caspase-3 and caspase-12, while autophagy activation (RAPA cotreatment) alleviated Cis-induced SGN apoptosis, reflected by downregulated TUNEL positive puncta, cleaved caspase-3 and caspase-12. Briefly, autophagy protect against Cis-induced apoptosis in SGNs *in vitro*.

### FAM134B-mediated ER-phagy was influenced by Cis-induced ROS accumulation

**FIGURE 7:**
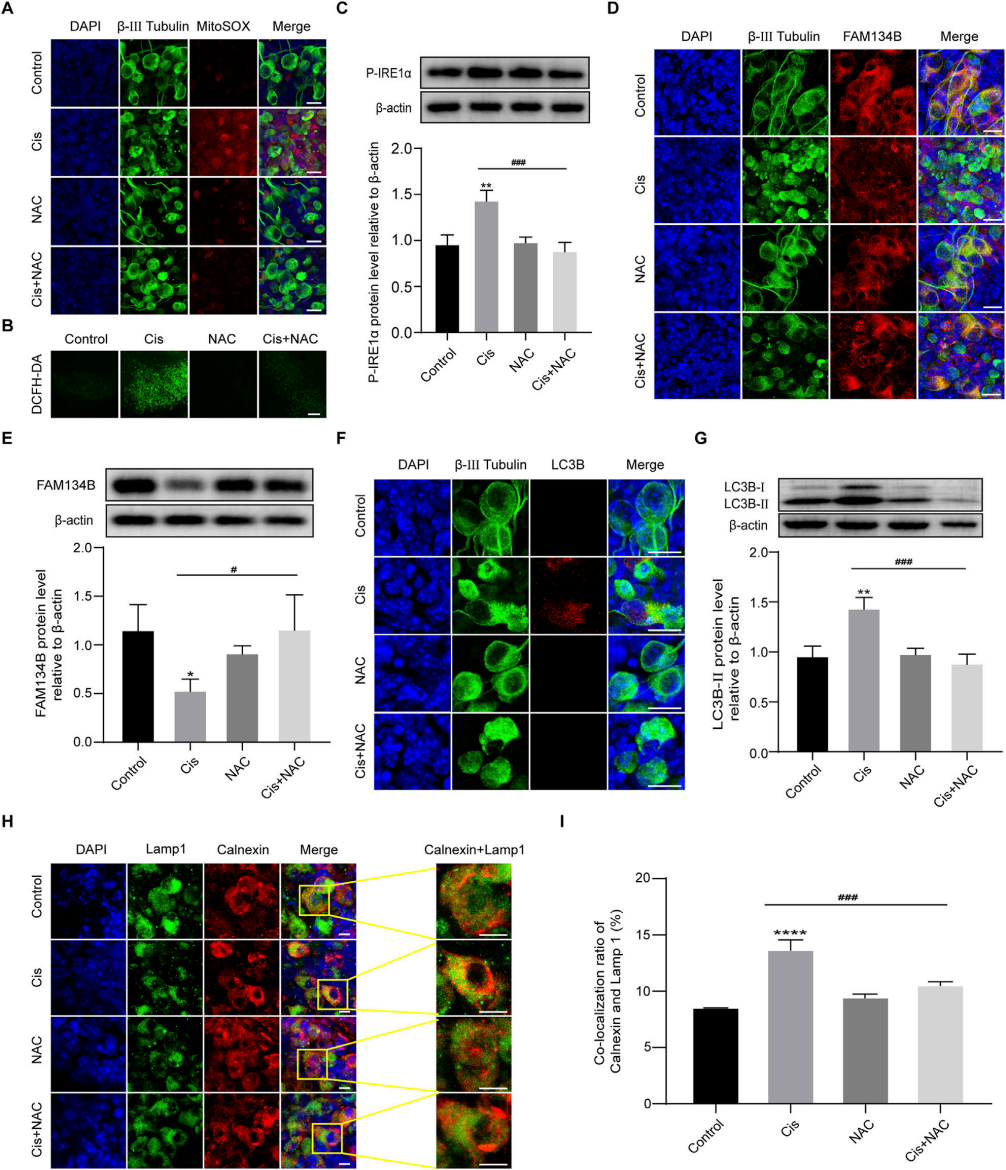
FAM134B-mediated ER-phagy was influenced by Cis-induced ROS accumulation. **(A, B)** ROS levels in SGNs were detected by MitoSOX Red (red) and DCFH-DA probe (green). Cis treatment led to obvious ROS accumulation in SGNs, which could be significantly alleviated by co-treatment of NAC. Scale bars: 15 µm in MitoSOX Red staining, 50 µm in DCFH-DA staining. **(C)** Changes of P-IRE1α in different groups were detected by WB, n = 3. **(D, E)** Co-staining of FAM134B (red) and β-III tubulin (green). Immunofluorescence showed that the expression of FAM134B was elevated in SGNs co-treated with NAC compared to Cis-only group, which was verified by WB. Scale bars = 15 μm, n = 3. **(F)** Double-staining of LC3B (red) and β-III tubulin (green). Scale bars = 15 µm. **(G)** WB showed the expression of LC3B-II in different groups, n = 3. **(H, I)** Fluorescence colocalization of Lamp1 (green) with Calnexin (red). The degree of colocalization was lower in the Cis + NAC group than in Cis-only group. Scale bars = 5 μm, n = 3. ^#^p < 0.05, ^###^p < 0.001 vs. cis group, *p < 0.05, **p < 0.01, ****p < 0.001 vs. control group.

In the mechanism of Cis-induced ototoxicity, including SGNs, the accumulation of ROS is the key factor to induce apoptosis (Nassauer et al., 2024). However, the correlation of FAM134B-mediated ER-phagy and ROS in Cis-induced SGN damage was unclear. Therefore, MitoSOX Red and DCFH-DA were used to assess the mitochondrial ROS and intracellular ROS levels in SGNs. What’s more, NAC, a powerful ROS inhibitor, was introduced to regulate the ROS levels in SGNs, and the treatment used here was based on our previously published study (Yang et al., 2018). The control group and NAC group showed a very low ROS level, which could be obviously enhanced in the Cis group, demonstrating the ROS accumulation in SGNs induced by Cis. Subsequently, we applied 2 mM NAC to pretreat SGNs for 2 h, then cotreat together with Cis for 24 h, which led to effective inhibition of ROS level compared with Cis-only group (Figures 7A, B). Moreover, we found that Cis + NAC co-treatment inhibited the expression of P-IRE1α and promoted the accumulation of FAM134B compared with Cis-only group (Figures 7C–E). In addition, results showed decreased level of both total autophagy and ER-phagy in ROS inhibiting (Cis + NAC) group, illustrated by decrease of LC3B (Figures 7F, G) and co-localization of lysosomes (Lamp 1) and ER (Calnexin) (Figures 7H, I). The results indicated a correlation between ROS, ER stress and ER-phagy, and, that is, higher ROS levels corresponds to increased ER stress and ER-phagy, and lower ROS levels corresponds to decreased ER stress and ER-phagy. All the data suggests that ROS plays an upstream role in Cis-induced SGN damage by influencing ER stress and ER-phagy, in which process FAM134B was involved.

### Inhibition of oxidative stress reduced apoptosis

**FIGURE 8:**
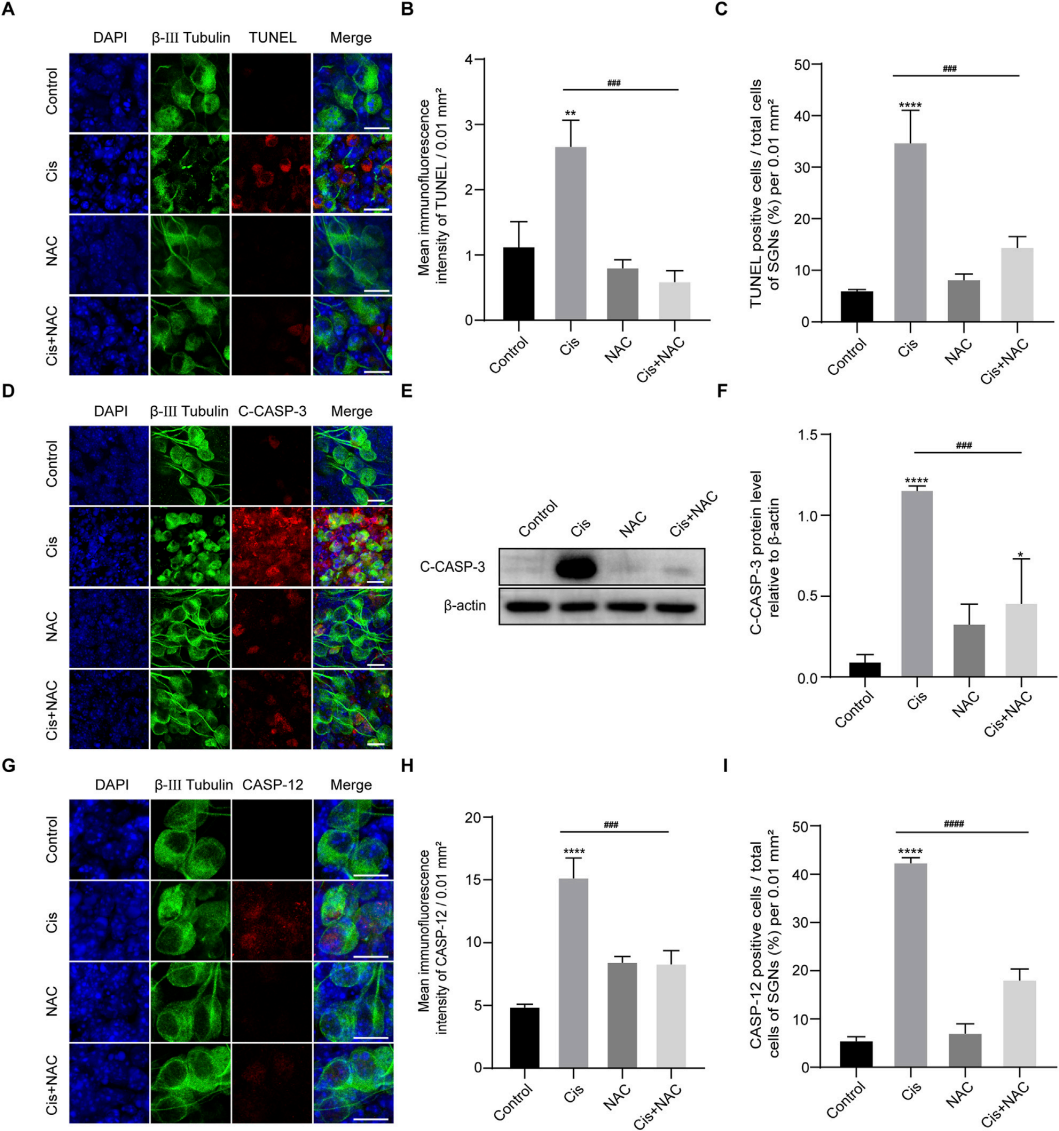
Inhibition of oxidative stress reduced apoptosis. **(A–C)** Representative images and analyses of TUNEL staining (red fluorescence) in SGNs. The amount of TUNEL-positive cells decreased after co-treatment with Cis and NAC. Scale bars = 15 μm, n = 3. **(D)** Co-staining of cleaved caspase-3 (red) and β-III tubulin (green). Cleaved caspase- 3 expression was cut down in the Cis + NAC group compared to the Cis group. Scale bars = 15 µm. **(E, F)** WB and quantitative analysis of cleaved caspase-3, n = 3. **(G–I)** Co-staining of caspase-12 (red) and β-III tubulin (green). Caspase-12 was significantly increased by Cis stimulus, which was decreased after inhibition of ROS in Cis + NAC group. Scale bars = 15 μm, n = 3. The differences were statistically significant. ^###^p < 0.001, ^####^p < 0.0001 vs. cis group, **p < 0.01, ****p < 0.001 vs. control group.

We then detected the effect of ROS on the final SGN fate by use of TUNEL assay. Results showed that the TUNEL-positive cells in the Cis + NAC group were significantly decreased than that in the Cis-only group (Figures 8A–C). Meanwhile, following experiments showed decreased expression of cleaved caspase-3 (Figures 8D–F) and caspase-12 (Figures 8G–I) after co-stimulated with NAC than treated with Cis only, which were main mediators of mitochondrial and ER related apoptotic pathway respectively. Results showed that the level of SGN apoptosis was in accordance with the ROS level in Cis injury.

### Knockdown the expression of FAM134B made SGNs more vulnerable to cisplatin-induced injury

**FIGURE 9:**
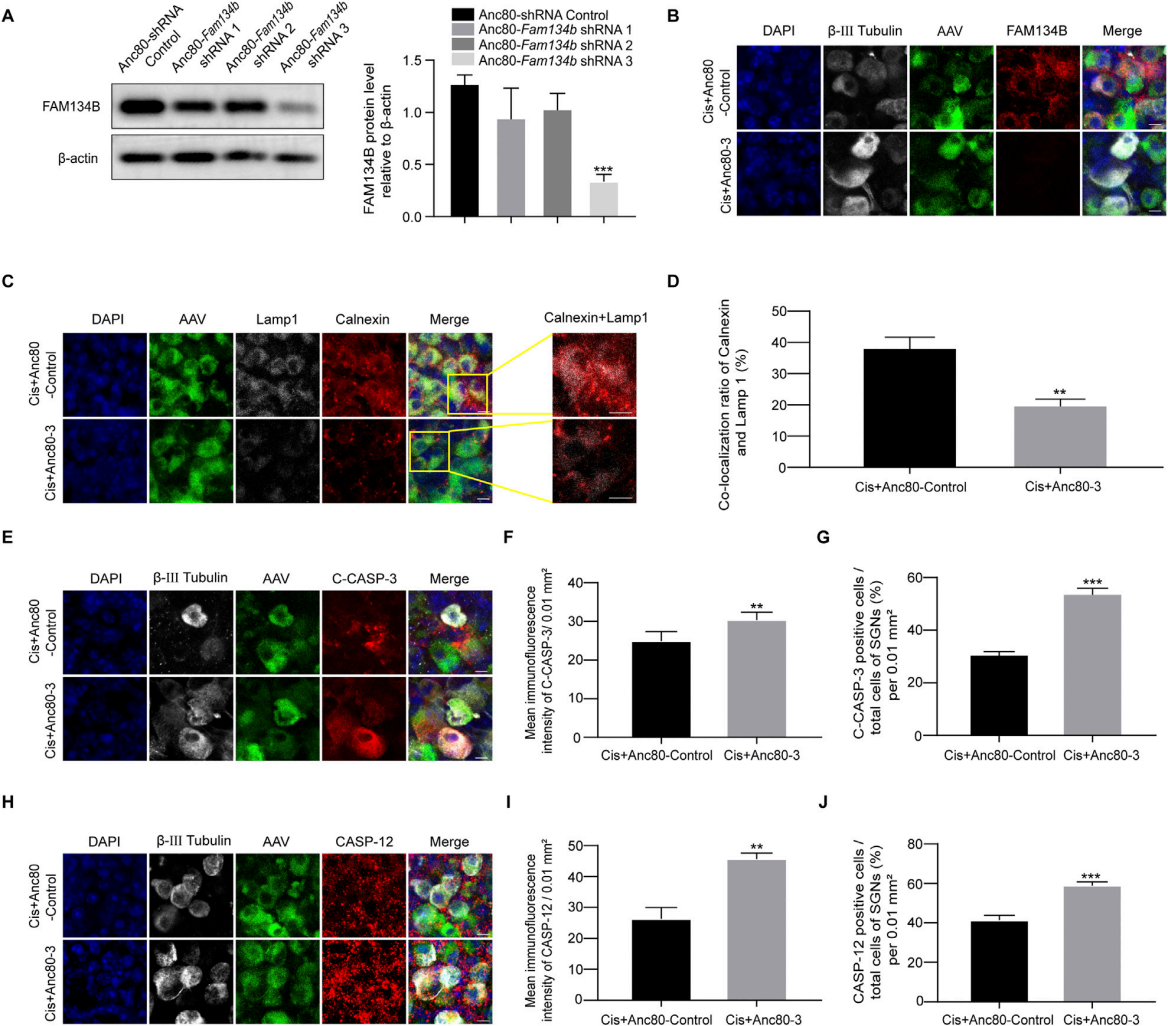
The damage of SGN was aggravated after FAM134B knockdown by AAV. **(A)** Western blot showed the expression of FAM134B after the three adenoviral sequences been transferred into the SGN, n = 3. **(B)** Immunofluorescence of FAM134B (red) and β-III tubulin (white) showed a marked decreased FAM134B puncta in SGNs after cisplatin treatment for 24 h with Anc80-*Fam134b*-shRNA-GFP-3 (Cis + Anc80-3) (green) compared to Cis + Anc80-shRNA-GFP-Control (Cis + Anc80-Control) group. Scale bars = 5 µm. **(C)** Fluorescence colocalization of LAMP1 (white) with Calnexin (red). Scale bars = 5 µm. **(D)** Proportion analysis of fluorescence co-localization, n = 3. **(E)** Co-staining of cleaved caspase-3 (red) and β-III tubulin (white) revealed variation of cleaved caspase-3 expression in SGNs collected from different groups. Scale bars = 5 µm. **(F, G)** Analysis of cleaved caspase-3 (red) in SGNs (β-III tubulin positive, white) after FAM134B knockdown by AAV. **(H–J)** Co-staining immunofluorescence of caspase-12 (red fluorescence) and β-III tubulin (white fluorescence). Immunofluorescence intensity of the caspase-12 and the number of caspase-12 positive SGN cells increased after FAM134B knockdown. Scale bars = 5 μm, n = 3. **p < 0.01, ***p < 0.001 vs. Cis + Anc80-Control or Cis + Anc80-3 groups.

We finally conducted *Fam134b* specific knockdown Anc80-*Fam134b*-shRNA-GFP-3 (Anc80-3) to make sure the true function of FAM134B in SGNs during cisplatin stimulus. Knockdown the expression of FAM134B in SGNs made a decrease of ER-phagy, labelled by the colocalization of LAMP1 with Calnexin, and an increase of apoptotic levels, marked by cleaved-caspase 3 and caspase 12 (Figure 9). Results verified the protective function of FAM134B mediated ER-phagy in SGNs against cisplatin-induced damage.

## Discussion

In this work, we demonstrated, for the first time, that FAM134B highly was expressed in the cytoplasm of neonatal (postnatal day 4, P4) C57BL/6 murine cochlear SGNs, followed by a sharp decrease as mice grew up, indicating the hypothesis of the important regulation of FAM134B in the early postnatal development of SGNs before hearing development, though the underlying mechanism needs to be further explored. In the subsequent experiments, the P4 mice were used for probing into the role of FAM134B in Cis-induced SGN damage *in vitro* for their high FAM134B expression in SGNs with no intra cochlear ossification, since the deep location of SGNs in the ossified temporal bone poses a significant challenge in entering and acquiring high-quality cochlear explants.

In the current study, we observed the fragmentation and lysis of nuclei, cellular atrophy, an increase in nerve fiber breaks, and a decrease in SGN cell density following Cis exposure (Figure 2), implying the successful construction of a Cis-related SGN damage model in P4 mice *in vitro*. Furthermore, the extent of SGN damage caused by Cis aggravated as the drug concentration increased and the exposure time prolonged, suggesting a concentration- and time-dependent manner of Cis-elicited SGN toxicity.

In this work, results demonstrated the elevation of TUNEL-positive SGNs in Cis group, which was at a much lower level in control group, revealing the apoptotic pathway in Cis-induced SGN injury, which is in consistence with findings from previous studies (Liu et al., 2021). The above work provided a solid basis for further investigation of the possible role of FAM134B in Cis-related SGN damage. In order to ascertain the specific apoptotic process, we subsequently conducted experiments to assess the pivotal apoptotic mediators, namely, bcl-2, caspase-3 and caspase-12. The activation of caspase-3, coupled with diminished bcl-2, signified the involvement of mitochondrial apoptotic pathway (Wang et al., 2022) in SGNs under Cis stimulus. Furthermore, the increased expression of caspase 12 indicated the involvement of another apoptotic pathway, ER-stress-mediated apoptosis (Szegezdi et al., 2003). Here, we investigated the two apoptotic mechanisms for reasons of established clues of tight interplay and cross talk between ER and mitochondria (Killackey et al., 2023), revealing responding of SGNs to Cis stress as a whole. These findings provided a foundation for exploration into the potential cross talk between FAM134B, mitochondrial and ER-related apoptosis in Cis-induced SGN damage. Subsequently, our attention shifted to the expression of FAM134B during Cis injury process. In the control group, we observed a significant high FAM134B expression in the cytoplasm of SGNs, which could be obviously diminished by treatment of Cis. Moreover, as the concentration of Cis increased and treat time prolonged, the expression of FAM134B gradually declined, indicating the effect of Cis on FAM134B in SGNs also has a concentration- and time-dependent manner. To try to uncover the reasons of the decline of FAM134B, we next measured autophagy, the most famous regulatory function of FAM134B (Mo et al., 2020). We find an obvious increase of LC3B and Lamp1 and Calnexin -double labeled puncta, indicating the activation of ER-phagy in Cis damage in SGNs. We concluded from the above results that the decreased expression of FAM134B was a result of activation of ER-phagy, leading to the elimination of itself, at least to some degree, together with the damaged ER fragments. So far, our data gave strong evidence of the involvement of FAM134B mediated ER-phagy in Cis-induced SGN damage.

We then wanted to know what led to FAM134B mediated ER-phagy, and how this happened during the Cis injury process in SGNs. Previous study have shown the regulatory role of ER stress in ER-phagy, and could be a powerful reason in fluoride-induced neurotoxicity (Niu et al., 2018). However, the relationship between FAM134B-mediated ER-phagy and ER stress in Cis-induced SGN damage remains unexplored. So, our focus was directed towards ER stress. IRE1α, an ER stress sensor, can be activated to maintain cellular proteostasis in response to ER stress (Wadgaonkar and Chen, 2021). Our findings demonstrated an increase in the activated phosphorylated form of IREα, known as P-IREα (Jin et al., 2021), following Cis stimulus, confirming the existence of ER stress in SGNs, which may be a powerful cause of FAM134B-mediated ER-phagy. And, the SGN apoptosis detected above may be a result of the persistent, unresolved and excessive ER stress, as prolonged ER stress has been verified to promotes cell death by apoptosis (Fernández et al., 2015). Collectively, the current findings indicated the involvement of ER stress, FAM134B mediated ER-phagy, and apoptosis in the context of Cis-induced SGN damage. However, the specific role of FAM134B-mediated ER-phagy, as well as its correlation with ER stress and apoptosis, remains uncertain.

In order to investigate the potential role of FAM134B-mediated ER-phagy in Cis-induced SGN damage, we sought to modulate autophagy by use of agonist, RAPA, and inhibitor, 3-MA (Zeng et al., 2023). Results revealed an increase of both total autophagy and ER-phagy, verified by elevated LC3B and Lamp1 and Calnexin -double labeled puncta ratio of ER and lysosomes, in Cis + RAPA group, and a decrease in the Cis + 3-MA group, compared to Cis-only group, suggesting the successful and effective regulation of autophagy, including ER-phagy. Next, we measured the expression of FAM134B and ER stress. Notably, our data revealed an increase of FAM134B and P-IRE1α in autophagy-inhibited group (Cis + 3-MA group), which may be a consequence of inhibited ER-phagy, leading to the decrease of clearing, while, on the other hand, the Cis stress was still existing, leading to the persistent induction of both ER stress and FAM134B. Not surprisingly, we obtained the contrary changes in the autophagy-activated group (Cis + RAPA group). Meanwhile, the increased apoptotic levels in the autophagy inhibitor group (Cis + 3-MA group) and the mitigated apoptotic levels in the autophagy activator group (Cis + RAPA group), proved the protective role of autophagy, FAM134B mediated ER-phagy included, in preventing SGN apoptosis induced by Cis. These results aligned with previous research demonstrating the protective function of FAM134B mediated ER-phagy in inhibiting neuronal apoptosis (Yao et al., 2023).

Furthermore, we investigated the potential causes of Cis-induced ER stress and FAM134B-mediated ER-phagy in SGNs. We conducted experiments to explore ROS, which has been previously identified as a key initiating factor in Cis-induced SGN damage in our published work (Yang et al., 2018). Observations revealed a significant increase in ROS levels in SGNs following Cis treatment, confirmed by the heightened fluorescence intensity of both MitoSOX and DCFH-DA, two commonly used ROS probes (Huang et al., 2023). To further validate the potential relationship between FAM134B-mediated ER-phagy and ROS, we employed NAC, an antioxidant that effectively reduced the ROS induced by Cis (Kim et al., 2023), in subsequent experiments. We measured decreased ER stress, FAM134B-mediated ER-phagy and apoptosis after the inhibition of ROS in Cis + NAC group compared with Cis-only group, verifying the accumulation of ROS was a key damage factor which initiated ER stress, FAM134B-medaited ER-phagy, and finally caused SGN apoptosis during the process of Cis-induced SGN damage.

Last but not least, we conducted *Fam134b* specific interfering AAV and make sure the protective function of FAM134B mediated ER-phagy in SGNs against cisplatin-induced damage.

In conclusion, the current study shows, for the first time, that FAM134B is expressed in the cytoplasm of SGNs, particularly in P4 C57BL/6 mice, suggesting that FAM134B might play a crucial role in the early postnatal development of SGNs prior to the development of hearing. Importantly, our investigation reveals the involvement of FAM134B-mediated ER-phagy in response to Cis-induced ROS accumulation and ER stress. Moreover, we revealed the protective function of FAM134B mediated ER-phagy in SGNs against cisplatin-induced damage. Consequently, our findings shed new light on the underlying mechanism of Cis-induced damage to SGNs and propose a promising therapeutic approach in mitigating sensorineural deafness.

## Data Availability

The raw data supporting the conclusions of this article will be made available by the authors, without undue reservation.
