# Supplementary figures and images for: FAM134B-mediated endoplasmic reticulum autophagy protects against cisplatin-induced spiral ganglion neuron damage
Source: Front Pharmacol. 2025 Jan 30;15:1462421. doi: 10.3389/fphar.2024.1462421 (PMC11821923; doi:10.3389/fphar.2024.1462421)

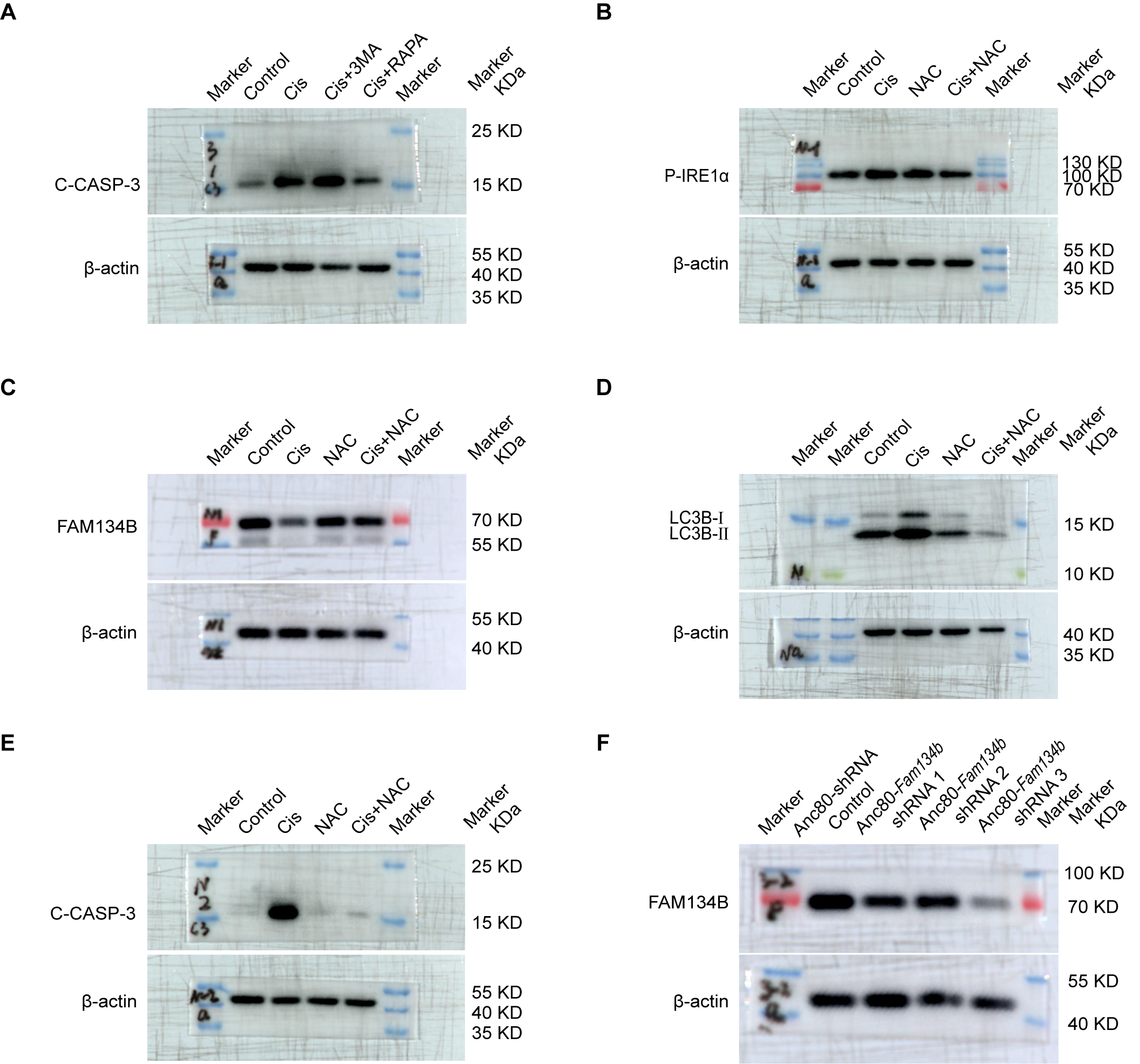

Supplement: Supplementary file 1 [file Image2.tif]

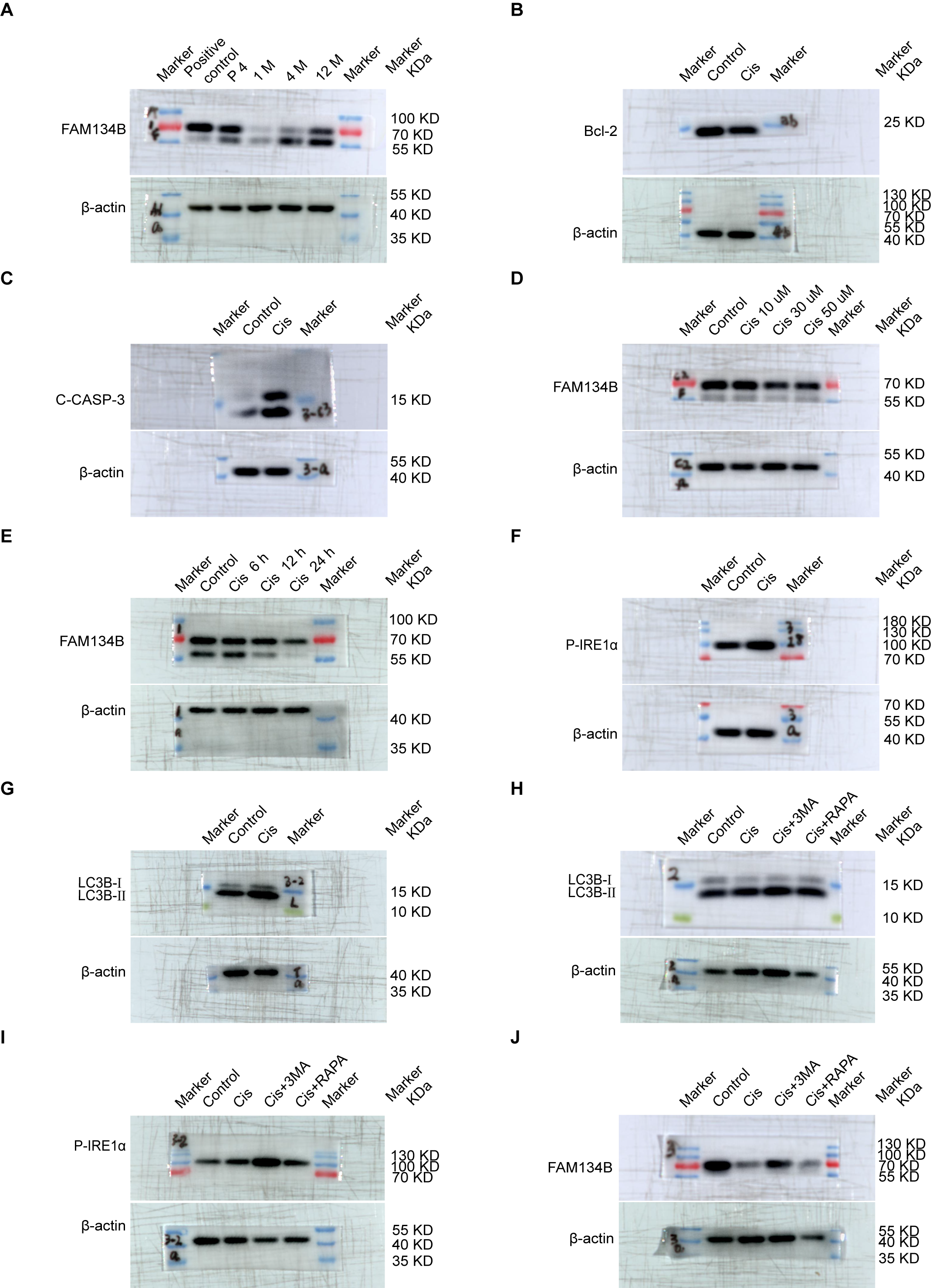

Supplement: Supplementary file 2 [file Image1.tif]
